# Gut commensal bifidobacteria are associated with restrained inflammatory reprogramming in human monocyte-derived dendritic cells

**DOI:** 10.1016/j.isci.2026.116583

**Published:** 2026-06-30

**Authors:** Sayaka Ishihara, Tatsuki Nishimura, Riko Mishima, Eri Mitsuyama, Akira Sen, Hiroki Kaneko, Yukihiro Hishida, Miyuki Tanaka, Toshitaka Odamaki

**Affiliations:** 1 Biotics Research Institute, R&D Division, Morinaga Milk Industry Co., Ltd., 5-1-83 Higashihara, Zama City, Kanagawa 252-8583, Japan; 2 Food Function Research Institute, R&D Division, Morinaga Milk Industry Co., Ltd., 5-1-83 Higashihara, Zama City, Kanagawa 252-8583, Japan

**Keywords:** innate immunity, innate memory, tolerance, microbiota, bifidobacteria

## Abstract

The gut commensal microbiota helps calibrate systemic innate immunity in humans. Here, we investigated whether gut bifidobacteria are associated with altered inflammatory responsiveness in human monocyte-derived dendritic cells (moDCs) from healthy adults. Higher abundance of gut-resident *Bifidobacterium* correlated with lower moDC inflammatory responses to multiple bacterial stimuli. *In vitro*, priming monocytes with Bifidobacterium before differentiation into moDCs induced transcriptomic and H3K4me1-associated chromatin remodeling and reduced inflammatory cytokine production upon rechallenge, while preserving the induction of selected phenotypic markers. These findings support an association between commensal bifidobacteria and restrained inflammatory reprogramming in human moDCs, characterized by attenuated inflammatory output without global loss of responsiveness under the conditions tested. Although our cohort and *in vitro* analyses do not establish causality, their convergence supports the biological plausibility of bifidobacterial modulation of monocyte-derived cell responses.

## Highlights

• Gut *Bifidobacterium* abundance associates with lower inflammatory moDC responses
• *Bifidobacteriu*m priming attenuates inflammatory responses upon rechallenge
• *Bifidobacterium*-primed moDCs retain phenotypic responsiveness after LPS rechallenge
• RNA-seq and H3K4me1 remodeling accompany restrained inflammatory responses

## Graphical abstract

**Figure:**
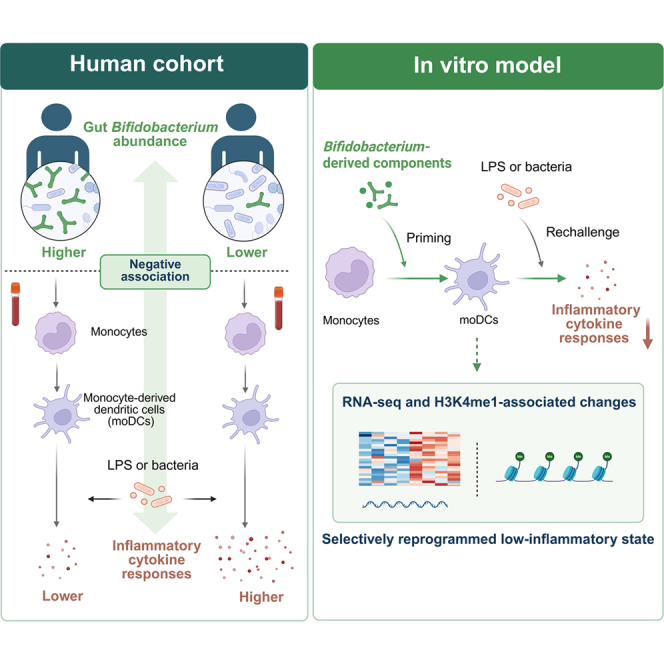

## Teaser

Epigenetics; Immunology; Systems biology

## Introduction

The gut microbiota maintains a mutually beneficial relationship with the host, contributing to homeostasis through the development, maturation, and regulation of the immune system. Conversely, dysbiosis triggered by antibiotics, diet, stress, and aging can disrupt this homeostatic relationship and contribute to conditions such as allergies, inflammatory bowel disease, and chronic inflammation.^1^^,^^2^ Although the gut microbiota is spatially confined to the intestinal lumen, recent studies suggest microbial products and metabolites can disseminate beyond the gut and influence systemic immunity.^3^^,^^4^ For instance, peptidoglycan, a major bacterial cell-wall component, has been detected in multiple tissues within 6 h of oral administration in mice.^5^ These disseminated microbial molecules and metabolites are increasingly recognized as important regulators of host defense and the maintenance of homeostasis. In addition to shaping adaptive immunity, including microbiota-specific T cells,^6^^,^^7^ systemic effector and memory T cell responses,^8^^,^^9^ CD8^+^ T cell functions,^10^ B-cell maturation,^11^ and systemic immunoglobulin regulation,^12^^,^^13^ the microbiota also influences bone marrow hematopoiesis,^14^^,^^15^ neutrophil functions,^16^ and activities of monocytes, macrophages, and dendritic cells (DCs).^17^^,^^18^^,^^19^^,^^20^ Nonetheless, much of our understanding derives from inbred mouse models, leaving the extent of systemic communication between the gut microbiota and human immune cells comparatively unexplored.

Because circulating immune cells represent a practical model for assessing how gut microbes influence innate immunity, investigations have focused on factors shaping the diversity of human innate immune responses. Indeed, emerging evidence indicates the gut microbiota modulates the production of inflammatory cytokines by peripheral blood mononuclear cells (PBMCs) and monocyte-derived dendritic cells (moDCs) upon microbial stimulation.^21^^,^^22^^,^^23^^,^^24^ Despite this, the specific gut microbial taxa and the mechanisms underlying such regulation of innate immune responses remain to be elucidated.

Here, we addressed these questions by examining the relationships between gut microbiota composition and innate immune responses to microbial stimulation in 50 healthy adults. We found that moDCs responded to four microbial stimuli—heat-killed *Bifidobacterium longum*, *Bifidobacterium breve*, *Lacticaseibacillus paracasei*, and lipopolysaccharide (LPS) from *Escherichia coli*—yet exhibited marked inter-individual variation in inflammatory cytokine production. Among the gut microbial taxa examined, *Bifidobacterium* abundance was negatively associated with inflammatory responsiveness, prompting us to further test whether bifidobacterial exposure could alter myeloid-cell responsiveness *in vitro*.

## Results

### Participant characteristics

In this study, of the 84 individuals screened, 60 met the inclusion criteria, and 50 were analyzed (Figure S1A). The study population consisted of healthy adults, including 32 males and 18 females, with a mean age of 39.9 ± 11.7 years and a mean BMI of 21.5 ± 2.1 (Table S1).

### Individual differences in moDCs' inflammatory responsiveness to bacterial stimulation

**Figure 1:**
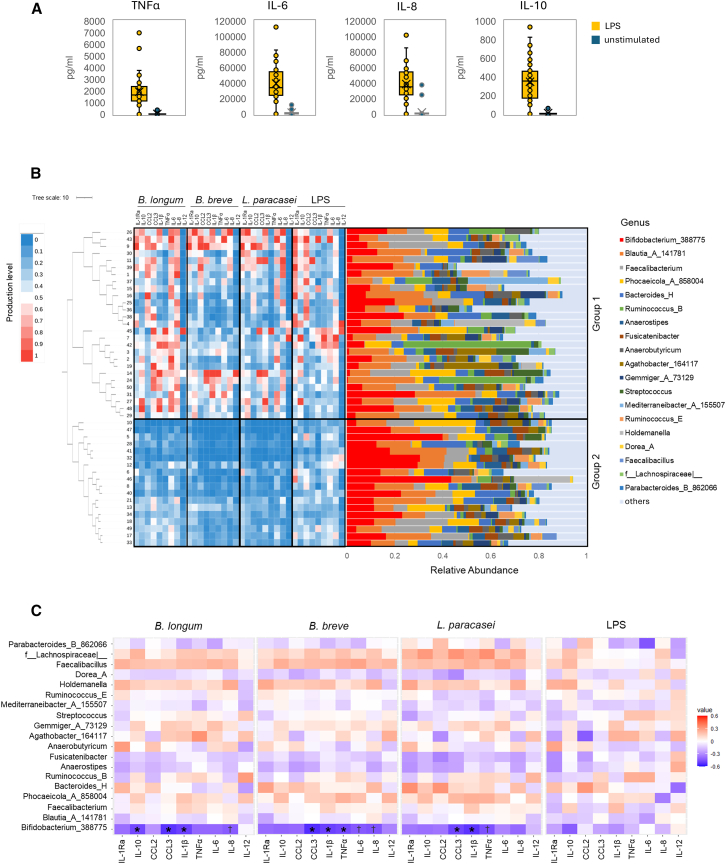
Gut *bifidobacterium* abundance is associated with lower cytokine responsiveness in human moDCs (A) Raw cytokine production by moDCs in response to 24 h stimulation with LPS. TNFα, IL-6, IL-8, and IL-10 are shown as representative cytokines. Unstimulated controls are shown for comparison. Each dot represents one participant; boxplots show the median and interquartile range. Statistical analysis for the full cytokine panel is provided in Figure S1B. (B) (left) Heatmaps with hierarchical cluster dendrograms. Columns represent participants, and rows represent cytokines/chemokines produced by moDCs in response to 24 h stimulation with *B. longum*, *B. breve*, *L. paracasei*, or LPS. Production levels are normalized, with the color scale indicating the magnitude (blue, low; red, high). Hierarchical clustering based on the Manhattan distance grouped participants into high-responder (Group 1) and low-responder (Group 2) categories. (right) Stacked bar plots show the genus-level gut microbiota composition of the corresponding participants. Less abundant taxa with a mean relative abundance <1% are grouped as “others.” Where genus-level resolution was not achievable, taxa are labeled at the lowest assigned taxonomic rank (i.e., f = family). (C) Spearman’s rank correlation analysis between cytokine concentrations in moDC culture supernatants under each stimulation condition and the relative abundance of bacterial genera (genus-level taxa with mean relative abundance >1%). Colors represent effect size (red: positive correlation; blue: negative correlation). Data are shown for *B. longum* (*n* = 50), *B. breve* (*n* = 49), *L. paracasei* (*n* = 48), and LPS (*n* = 45). Statistical significance was assessed using two-sided Spearman’s rank correlation with Benjamini-Hochberg correction for multiple testing and is indicated in the heatmap (†*p* < 0.1 and ∗*p* < 0.05). Correlation coefficients and corresponding raw and adjusted *p* values are provided in Table S4.

To assess interindividual variation in direct innate immune responses to bacteria, we generated moDCs from PBMCs and stimulated them for 24 h with one of three heat-killed Gram-positive bacteria *(B. longum, B. breve, and L. paracasei*)—commonly found in the human gut or used as probiotics and reported to have immunomodulatory properties—or LPS, a representative Gram-negative-derived stimulus. These stimuli were selected to provide a defined panel of Gram-positive bacterial and LPS challenges for standardized comparison of inflammatory responsiveness across participants, rather than to recapitulate the most abundant taxa detected in each individual microbiome. We measured a predefined panel of DC-related cytokines and chemokines spanning inflammatory, regulatory, and chemotactic readouts (IL-1Ra, IL-10, CCL2, CCL3, IL-1β, TNFα, IL-6, IL-8, and IL-12). All stimuli elicited higher cytokine levels compared with unstimulated controls, although the magnitude varied substantially among individuals (Figures 1A and S1B). The extent of cytokine induction differed by stimulus, with *B. longum* inducing the highest levels for most cytokines (Figure S1B). Certain participants consistently demonstrated high cytokine responses (Group 1), whereas others showed lower responses (Group 2), regardless of the stimulus (Figure 1B). These findings indicate that moDC inflammatory responsiveness was broadly consistent across distinct bacterial stimuli within individuals, while exhibiting clear interindividual differences.

### Gut bifidobacterium abundance is associated with lower inflammatory responsiveness

We next explored factors potentially contributing to these interindividual differences, focusing on host characteristics previously linked to innate immune variation, including sex,^25^ age,^26^ diet,^27^ and the gut microbiota composition.^3^ Consistent with previous findings,^24^ sex and age showed no clear or consistent associations with cytokine responses across stimuli, although a small number of nominal associations were observed (Table S2). Exploratory analyses of dietary variables derived from the BDHQ similarly revealed no clear or consistent associations with moDC cytokine responses (Table S3).

Table S2. Background factors and cytokine responses, Related to Figure 1

Table S3. BDHQ and cytokine responses, Related to Figure 1

By contrast, gut microbiota composition varied across participants and showed differences in its overall structure when comparing high- and low-responder groups (Figure S1C). Among the 412 genera detected, 19 exhibited mean relative abundance exceeding 1%, with *Bifidobacterium*_388775 (genus-level annotation) being the most abundant (13.1% ± 10.2%) (Figure 1B). Correlation analysis showed that the relative abundance of *Bifidobacterium*_388775 was negatively correlated with inflammatory cytokine responses, with correlation coefficients reaching approximately −0.4 for those cytokines showing statistically significant associations (Figure 1C and Table S4). Although not all cytokines reached statistical significance, *Bifidobacterium* was the only genus that retained significant associations after multiple testing correction. Several other taxa, including *Lachnospiraceae*, *Faecalibacillus*, and *Holdemanella*, showed weak positive trends, but these did not remain significant after correction (Figure 1C and Table S4).

Table S4. 16S rRNA gene–based microbiota composition and cytokine responses, Related to Figure 1

To refine these associations at higher taxonomic resolution, we performed shotgun metagenomic sequencing and re-evaluated the relationships at the species level. This analysis suggested that negative associations with inflammatory cytokine responses were not uniform across *Bifidobacterium* species. While *Bifidobacterium pseudocatenulatum* showed relatively stronger negative associations, *Bifidobacterium longum* exhibited the same directional trend, with differences in correlation strength observed across species (Figure S1D and Table S5). In contrast, standard diversity indices, including observed features, Shannon entropy, and Faith’s PD, showed little association with cytokine responsiveness (Table S2). Together, these findings indicate that lower inflammatory responsiveness in this cohort was associated with specific microbial taxa, including *Bifidobacterium*, and that the strength of this association may vary across *Bifidobacterium* species, rather than being explained by overall measures of microbial diversity.

Table S5. Shotgun metagenomic profiles and cytokine responses, Related to Figure S1

### Bifidobacterium priming attenuates inflammatory responses upon rechallenge in moDCs

**Figure 2:**
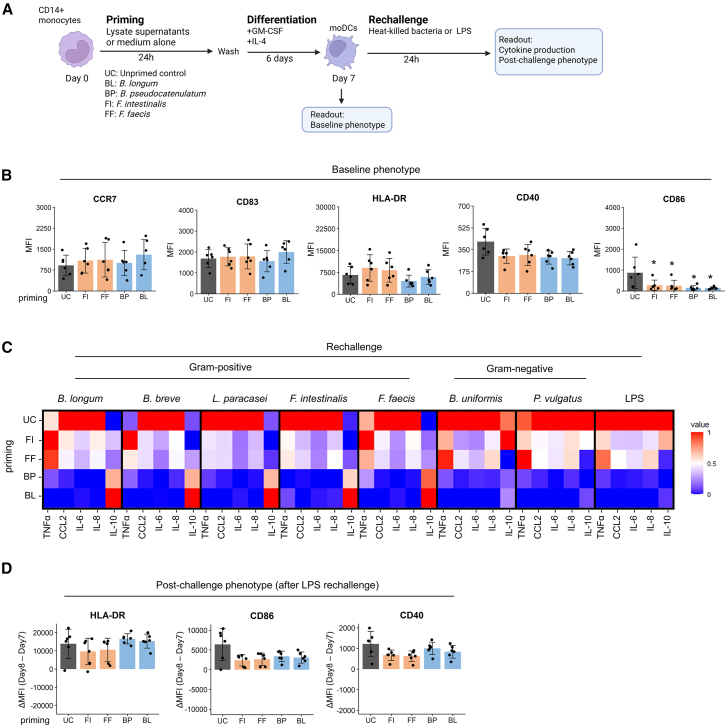
*Bifidobacterium* priming attenuates inflammatory cytokine responses upon rechallenge in moDCs (A) Schematic of the *in vitro* priming-differentiation-rechallenge model. Human monocytes were primed for 24 h with bacterial lysate supernatants (BL, BP, FI or FF), or with medium alone as an unprimed control (UC), washed, and then differentiated into moDCs in the presence of GM-CSF and IL-4 for 6 days. Differentiated moDCs were subsequently rechallenged for 24 h with heat-killed bacteria or LPS. Day 7 moDCs were used for baseline phenotypic analyses before rechallenge, whereas day 8 cells and supernatants were used for post-rechallenge analyses, including cytokine production and phenotypic analyses. The schematic was created with BioRender.com. (B) Baseline phenotypic status of day 7 moDCs prior to secondary rechallenge. Surface expression of CCR7, CD83, HLA-DR, CD40, and CD86 was assessed by flow cytometry across unprimed controls and bacterial lysate-primed conditions (*n* = 6). Data are shown as mean ± SD with individual data points. Statistical analysis was performed using Dunnett’s test with Benjamini-Hochberg correction for multiple testing; adjusted *p* values are indicated where applicable (∗adj. *p* < 0.05). (C) Heatmap of normalized cytokine and chemokine responses after rechallenge with the indicated secondary stimuli (n = 3–8). For each cytokine or chemokine, values were normalized to a 0–1 scale based on the minimum and maximum production levels observed across UC-, FI-, FF-, BP-, and BL-moDCs. Colors indicate relative production levels (blue, low; red, high). Fold-change data and statistical comparisons are shown in Figure S3. (D) Post-rechallenge phenotypic responses after LPS rechallenge, shown as changes in surface-marker expression (ΔMFI, calculated as day 8 minus pre-rechallenge day 7) for HLA-DR, CD86, and CD40 to assess the capacity for marker induction after rechallenge (*n* = 6). Data are shown as mean ± SD with individual data points. Statistical analysis was performed using Dunnett’s test with Benjamini-Hochberg correction for multiple testing; no statistically significant differences were observed among conditions.

Recent studies have shown that prior microbial stimulation can alter subsequent responsiveness in innate immune cells, including monocytes and moDCs.^28^^,^^29^^,^^30^ To experimentally follow up the cohort-based association under standardized *in vitro* conditions, we used a priming-differentiation-rechallenge framework to test whether bifidobacterial exposure could alter subsequent inflammatory responsiveness in human monocyte-derived cells. In this study, monocytes were primed with soluble bacterial components using lysate supernatants derived from live bacteria, differentiated into moDCs, and then rechallenged with heat-killed bacteria to assess secondary cytokine responsiveness (Figure 2A). Lysate supernatants were used for priming to assess the effects of soluble bacterial components while minimizing carryover of intact bacterial material into the subsequent differentiation and rechallenge phases. For priming, we selected two *Bifidobacterium* species (*B. pseudocatenulatum* and *B. longum*) based on their detection in the 16S rRNA gene profiling and negative trends in the shotgun metagenomic analysis. As non-*Bifidobacterium* comparator taxa, we included two *Faecalibacillus* species (*F. intestinalis* and *F. faecis*) to position the bifidobacteria-induced phenotype relative to taxonomically distinct commensal bacterial conditions within the same priming-rechallenge assay. These taxa also showed weak positive point estimates in the 16S analysis, although these associations did not reach statistical significance. For rechallenge, two *Bacteroides* species, abundant Gram-negative commensals in the human gut, were added to the initial four bacterial stimuli. In addition, two *Faecalibacillus* species were also included to assess homologous responses.

To determine whether bacterial priming affected moDC differentiation and baseline phenotypic status, flow-cytometric analysis was performed on unprimed control moDCs (UC-moDCs) and moDCs primed with lysate supernatants of *F. intestinalis* (FI-moDCs), *F. faecis* (FF-moDCs), *B. pseudocatenulatum* (BP-moDCs), and *B. longum* (BL-moDCs). Differentiation into moDCs at day 7 was supported by a substantial reduction in CD14 expression, induction of CD209, and preserved viability under all conditions (Figures S2A and S2B). We next examined the baseline phenotypic status of these cells before secondary stimulation. CCR7, CD83, HLA-DR, and CD40 showed no significant differences among conditions, whereas CD86 expression was reduced in primed moDCs relative to unprimed controls (Figure 2B).

We then assessed cytokine production after rechallenge with heat-killed bacteria or LPS. Upon rechallenge with the eight stimuli, BP-moDCs and BL-moDCs produced significantly lower levels of inflammatory cytokines compared with unprimed controls and non-*Bifidobacterium*-primed moDCs. In contrast, IL-10 did not follow the same uniform reduction pattern and appeared to vary depending on the secondary stimulus (Figures 2C and S3). As a reference condition for a low-responsive state, cytokine responses were also assessed in cells primed with LPS and rechallenged with LPS.^31^ Consistent with features described for LPS tolerance, LPS-primed moDCs showed significantly reduced inflammatory cytokine production compared with unprimed controls upon LPS stimulation (Figure S4A).

To determine whether bifidobacteria-primed moDCs remained responsive at the phenotypic level, changes in surface marker expression were assessed following secondary LPS challenge. LPS rechallenge increased HLA-DR, CD40, and CD86 expression across conditions (Figure 2D), indicating that bifidobacteria-primed moDCs retained phenotypic responsiveness despite reduced inflammatory cytokine production and were therefore not in a globally unresponsive or terminally refractory state.

To examine whether the observed effect extended beyond the two initially tested *Bifidobacterium* species, we expanded the analysis to include two additional species, *B. adolescentis* and *B. breve*, and evaluated them in the same experimental framework. All four *Bifidobacterium* species tested here showed a similar directional reduction in inflammatory cytokine production following secondary LPS stimulation compared with unprimed controls (Figure S4B). These results indicate that the attenuation of secondary inflammatory responses after *Bifidobacterium* priming is not limited to *B. pseudocatenulatum* and *B. longum* but is also observed in the additional *Bifidobacterium* species examined here. Moreover, the low-responsive phenotype induced by *Bifidobacterium* species showed a dose-dependent relationship (Figure S4C).

Finally, to assess whether the attenuation of inflammatory responses associated with *Bifidobacterium* priming extends beyond the monocyte stage, we administered *Bifidobacterium* lysate supernatants to fully differentiated moDCs. These primed moDCs exhibited significant reduction in inflammatory cytokine production upon rechallenge with LPS and *B. longum* (Figures S4D and S4E), indicating that this response-attenuating effect is also observed in already differentiated moDCs.

### Bifidobacterium priming reshapes the baseline transcriptional state of moDCs

**Figure 3:**
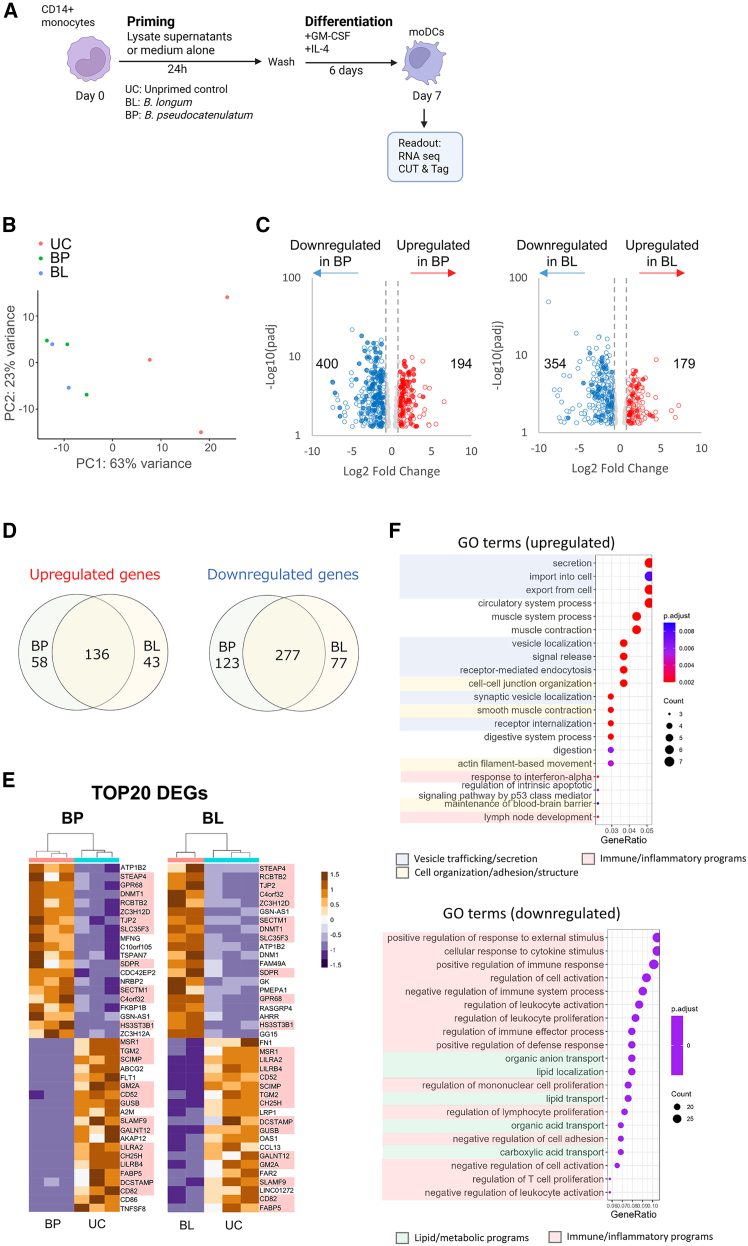
*Bifidobacterium* priming is associated with a distinct baseline transcriptional state of moDCs (A) Schematic of the sampling strategy for transcriptomic and epigenetic analyses. Monocytes were primed with BP or BL, or left unprimed as controls (UC), followed by differentiation into moDCs. Day 7 moDCs were collected prior to rechallenge and used for RNA-seq and CUT&Tag analyses. The schematic was created with BioRender.com. (B) Principal component analysis (PCA) of RNA-seq data from BP-, BL-, and UC-moDCs. Data are shown for BP-moDCs (*n* = 3), BL-moDCs (*n* = 2), and UC-moDCs (*n* = 3). (C) Volcano plots illustrating differentially expressed genes (DEGs) in BP- and BL-moDCs compared with UC-moDCs. Upregulated genes (log2 fold change [FC] > 1, adj. *p* < 0.05) are shown in red; downregulated genes (log2 FC < −1, adj. *p* < 0.05) in blue. Solid circles indicate genes shared by BP- and BL-moDCs; open circles indicate genes unique to one condition. (D) Venn diagram showing overlap of upregulated genes (left) and downregulated genes (right) between BP- and BL-moDCs. (E) Heatmap of the top 20 DEGs in BP- and BL-moDCs compared with UC-moDCs. Expression values are scaled from −1.5 to +1.5. Genes shared between BP- and BL-moDCs are shown in red. (F) Dot plots of the top 20 enriched Gene Ontology (GO) biological process terms among genes commonly upregulated (top) or downregulated (bottom) in BP- and BL-moDCs relative to UC-moDCs. Terms are grouped into broader functional categories. The gene ratio indicates the percentage of candidate genes within each pathway. Dot color indicates the adjusted *p* value (adj. *p* < 0.05), and dot size corresponds to the number of genes in each pathway.

To elucidate the transcriptional basis of the *Bifidobacterium*-associated low-inflammatory phenotype, we performed transcriptomic analysis on BL-moDCs, BP-moDCs, and UC-moDCs (Figure 3A), prioritizing the identification of molecular features shared within the *Bifidobacterium* group. Principal component analysis (PCA) demonstrated that the dominant variation in gene expression arose from *Bifidobacterium* priming, with the first principal component (PC1) separating BL-moDCs and BP-moDCs from controls (Figure 3B). Both BL-moDCs and BP-moDCs exhibited roughly twice as many downregulated genes as upregulated, and notably, over 70% of differentially expressed genes (DEGs) were shared between the two groups (Figures 3C and 3D). A similar overlap was observed among the top 20 DEGs (Figure 3E).

Gene ontology (GO) terms and KEGG pathway enrichment analyses of the overlapping DEGs revealed coordinated changes across multiple functional categories. Upregulated genes were enriched mainly in vesicle trafficking-, secretion-, and cell-organization-related processes, including vesicle localization, secretion, and cell-cell junction organization. In parallel, KEGG pathways enriched among upregulated genes were grouped predominantly into vesicle trafficking/secretion and cell organization/adhesion/structure modules (Figures 3F and S5A, and Table S6). By contrast, downregulated genes were enriched for immune/inflammatory and lipid/metabolic programs, including positive regulation of immune response, cellular response to cytokine stimulus, regulation of cell activation, lipid localization, and organic anion transport. Consistently, KEGG pathways enriched among downregulated genes clustered mainly into immune/inflammatory and lipid/metabolic modules, including PI3K-Akt signaling, cytokine-cytokine receptor interaction, chemokine signaling pathway, and PPAR signaling pathway (Figures 3F and S5A, and Table S6).

Table S6. RNA-seq data, Related to Figure 3

Together, these analyses indicate that *Bifidobacterium* priming is associated with selective transcriptional changes in moDCs, characterized by reduced immune/inflammatory and lipid/metabolic programs together with relative enrichment of vesicle trafficking, secretion, and cell-organization-related processes, rather than a global suppression of transcriptional activity.

### Bifidobacterium priming is associated with selective H3K4me1 remodeling in moDCs

**Figure 4:**
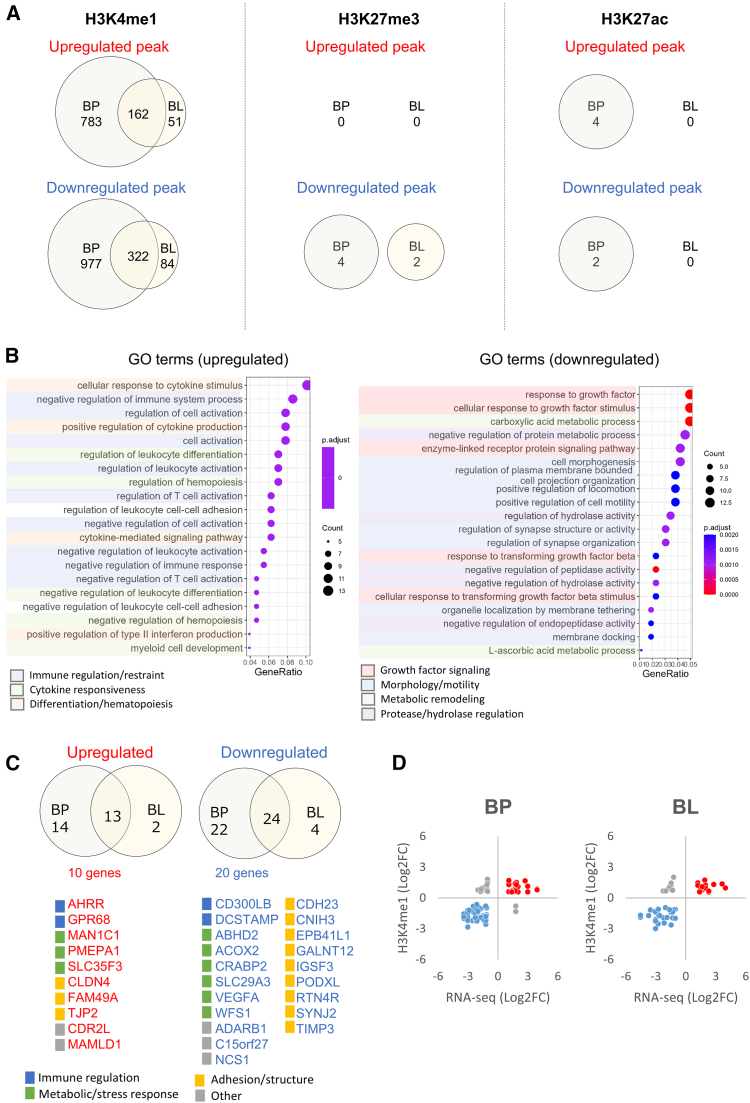
*Bifidobacterium* priming is associated with selective H3K4me1 remodeling in moDCs (A) Venn diagram showing differentially modified peaks in BP- moDCs and BL-moDCs relative to UC-moDCs for H3K4me1, H3K27me3, and H3K27ac. Upregulated and downregulated peaks are shown separately. Data are from BP-moDCs (*n* = 3), BL-moDCs (*n* = 2), and UC-moDCs (*n* = 3). Differential peaks were defined using an adjusted *p* < 0.05 and a log2 fold-change cutoff of ±0.5. (B) Dot plots of the top 20 enriched GO biological process terms derived from significantly altered H3K4me1 peaks shared by BP- and BL-moDCs relative to UC-moDCs. Terms are grouped into broader functional categories. Gene ratio, *p* value scale, and node sizes are shown as in Figure 3F. (C) Venn diagram summarizes integrated analysis of H3K4me1 (CUT&Tag) and gene expression (RNA-seq) data (adj. *p* < 0.05). Genes showing concordant changes in both H3K4me1 signal and gene expression in BP- and BL-moDCs are shown. Commonly altered genes were summarized at the unique-gene level for visualization and grouped by functional category; the full peak-gene associations are provided in Table S8. (D) Scatterplot correlating H3K4me1 peak changes with mRNA expression in BP- or BL-moDCs relative to UC-moDCs. Significantly upregulated (log2 FC > 0.5 for H3K4me1; log2 FC > 1 for gene expression; adj. *p* < 0.05) features are shown in red, and significantly downregulated (log2 FC < −0.5 for H3K4me1; log2 FC < −1 for gene expression; adj. *p* < 0.05) features in blue.

The shared transcriptional changes observed in BP-moDCs and BL-moDCs led us to examine whether *Bifidobacterium* priming was also associated with epigenetic remodeling. We therefore analyzed histone modifications associated with active (H3K4me1, H3K27ac) and repressive (H3K27me3) chromatin states in moDCs (Figures 3A and 4A). Relative to the unprimed control, priming with the two *Bifidobacterium* species induced prominent alterations in H3K4me1 marks in moDCs. In total, approximately 2300 regions of H3K4me1 marks were significantly changed in BP-moDCs, and approximately 600 regions in BL-moDCs (adj. *p* < 0.05; Table S7). Interestingly, only about 20% of these modifications overlapped in BP- vs. BL-primed cells (Figure 4A and Table S7), suggesting each *Bifidobacterium* species induces partially distinct H3K4me1-mediated epigenetic modifications. In contrast, H3K27ac and H3K27me3 marks exhibited few significant changes following *Bifidobacterium* priming (Figure 4A and Table S7).

Table S7. CUT&Tag data, Related to Figure 4

GO and KEGG enrichment analyses of genes near significantly altered H3K4me1 peaks also revealed coordinated changes across multiple functional categories. Upregulated H3K4me1-associated terms were enriched for immune regulatory processes, cytokine responsiveness, and differentiation/hematopoiesis-related processes, including negative regulation of immune system process, cellular response to cytokine stimulus, and myeloid cell development (Figure 4B and Table S7). By contrast, downregulated terms were enriched for growth factor signaling, morphology/motility, and metabolic remodeling, such as response to growth factor, cell morphogenesis, positive regulation of cell motility, and carboxylic acid metabolic process. KEGG analysis supported this pattern, with pathways associated with downregulated H3K4me1 peaks clustering mainly into lipid/metabolic programs (Figures 4B and S5B, and Table S7). Together, these analyses suggest that *Bifidobacterium* priming is associated with H3K4me1-centered epigenetic remodeling in moDCs, affecting immune-related, metabolic-related, and structure/state-related programs, with prominent changes in immune regulatory processes, cytokine responsiveness, differentiation/hematopoiesis, and metabolic remodeling.

Integrative analyses of H3K4me1 peak annotations and RNA-seq data showed that a subset of H3K4me1-associated genes exhibited concordant transcriptional changes in both BP-moDCs and BL-moDCs (Figures 4C and 4D, and Table S8). These concordant genes were grouped into three major functional categories: immune regulation, metabolic/stress-response pathways, and adhesion/structure-associated programs. Representative concordantly changed genes mapped across all three functional categories. These included AHRR and GPR68 in immune regulation, ACOX2, CRABP2, and VEGFA in metabolic/stress-response pathways, and TJP2, CLDN4, TIMP3, PODXL, and RTN4R in adhesion/structure-associated programs. Taken together, these data suggest *Bifidobacterium* priming is associated with selective overlap between H3K4me1 remodeling and transcriptional changes in genes related to immune regulation, metabolic/stress-response pathways, and adhesion/structure-associated programs.

Table S8. Integrated analysis data, Related to Figure 4

## Discussion

The gut microbiota is an important regulator of host immune homeostasis,^3^ but the specific intestinal bacteria that shape systemic innate immune responsiveness remain incompletely defined. Although commensal microorganisms are spatially confined to the gut lumen, microbial products and metabolites can influence peripheral immune cells and contribute to inter-individual variation in inflammatory responses.^21^^,^^22^^,^^23^ In this study, we identified an association between gut *Bifidobacterium* abundance and reduced inflammatory responsiveness of human moDCs, and further examined whether bifidobacterial exposure at the monocyte stage could modulate post-differentiation responsiveness *in vitro*.

Individuals with a greater abundance of *Bifidobacterium* showed lower moDC inflammatory responses to heat-killed Gram-positive bacteria and LPS. In parallel, priming of monocytes with lysates from gut-resident *Bifidobacterium* species attenuated inflammatory cytokine production upon subsequent Gram-positive and Gram-negative bacterial challenge and was accompanied by transcriptomic and H3K4me1-associated chromatin remodeling. Together, these findings suggest that greater gut *Bifidobacterium* abundance is associated with lower inflammatory responsiveness in human moDCs, and that bifidobacterial priming is associated with an attenuated inflammatory response state *in vitro*. Although direct causal relationships cannot be established from the cohort and *in vitro* analyses, both approaches pointed to a qualitatively similar phenotype characterized by broadly attenuated inflammatory responses across distinct stimuli, supporting the biological plausibility that bifidobacterial exposure can modulate inflammatory responsiveness in human monocyte-derived cells.

Bifidobacteria are common members of the human gut microbiota and have been linked to immune homeostasis in several settings.^32^^,^^33^ Previous studies have associated bifidobacteria with reduced allergic and inflammatory phenotypes,^34^^,^^35^^,^^36^^,^^37^^,^^38^^,^^39^ with proposed mechanisms including the induction of regulatory T cells,^40^^,^^41^ suppression of Th2- and Th17- associated responses,^42^^,^^43^ and modulation of innate immune cell functions, including those of DCs and macrophages.^32^^,^^44^ In addition, bifidobacterial surface-associated molecules, including exopolysaccharides and other cell-surface polysaccharides, have been reported to regulate host immune activation.^41^^,^^44^ Notably, some bifidobacterial components have been shown to suppress costimulatory molecule expression and DC-mediated T cell activation,^44^ suggesting that specific bifidobacterial signals can act to restrain excessive immune activation. Collectively, these studies support an immunomodulatory role for bifidobacteria, but how prior exposure shapes later responsiveness in innate immune cells remains incompletely understood. Our results extend this literature by suggesting that bifidobacteria can modulate the functional state acquired during the monocyte-to-moDC culture process and attenuate secondary inflammatory responsiveness in human monocyte-derived cells, in association with coordinated transcriptional and chromatin changes. In the present study, all four bifidobacterial species tested *in vitro* showed the same directional trend toward attenuated responsiveness in human moDCs, whereas the shotgun metagenomic analysis and H3K4me1-associated chromatin profiles of BP- and BL-moDCs suggested species-level differences in the strength and molecular features of this phenotype. Consistent with the known species- and strain-dependent immunomodulatory properties of bifidobacteria,^32^^,^^45^ these findings suggest that while reduced inflammatory responsiveness may be a shared qualitative feature, its magnitude and underlying molecular programs vary across taxa and warrant further investigation.

A key interpretive question is whether this low-responsive phenotype simply reflects enhanced dendritic-cell maturation before rechallenge. *Bifidobacterium*-primed cells did not show a consistent baseline increase in commonly used maturation- or migration-associated markers, and they retained the capacity to increase selected phenotypic markers, including CD86, CD40, and HLA-DR, after LPS rechallenge despite producing less inflammatory cytokine. Thus, the phenotype observed here is not readily explained by a uniformly mature or migratory dendritic-cell state alone, but is more consistent with selective functional reprogramming in which inflammatory output is attenuated despite preserved phenotypic responsiveness, rather than with a uniformly immunosuppressed or terminally mature state.

This interpretation is further supported by transcriptomic and H3K4me1-associated chromatin remodeling in bifidobacteria-primed moDCs. RNA-seq, which reflects the baseline transcriptional state after priming and differentiation, showed reduced immune/inflammatory programs together with changes in metabolic and metabolite-transport-related pathways, including lipid localization, organic anion transport, and PPAR-associated pathways. At the same time, genes associated with vesicle trafficking, secretion, cell-cell contact, and structural organization were relatively enriched. Thus, the transcriptional pattern does not indicate a uniform reduction in cellular programs, but rather a selective remodeling of the moDC baseline state, in which inflammatory and metabolic/stress-response programs are attenuated while trafficking, secretory, and cell-organization-related programs are relatively enriched. Given the established links between lipid metabolism and dendritic-cell activation and function,^46^^,^^47^ lipid-associated pathways may represent one relevant component of this altered response state, although their functional contribution to attenuated inflammatory responsiveness remains to be determined.

Beyond the baseline transcriptional changes, H3K4me1-associated remodeling may reflect chromatin states that influence responsiveness to subsequent stimulation rather than steady-state transcriptional output itself.^48^ Consistent with this, bifidobacterial priming induced extensive H3K4me1-associated chromatin remodeling, characterized by the enrichment of immune-regulatory and cell-state control processes and attenuation of growth- and motility-associated pathways. Given the association of H3K4me1 with enhancer priming and poised chromatin states, these changes may reflect a priming-associated chromatin landscape established by the initial stimulus that could contribute to shaping responsiveness upon secondary challenge.^48^^,^^49^

The limited overlap between these layers is consistent with the idea that only a subset of priming-induced chromatin changes is already reflected in steady-state transcription.^48^^,^^49^ Importantly, the concordantly changed genes were concentrated within functional axes related to immune regulation, metabolic/stress-response pathways, and adhesion/structure-associated programs, consistent with selective rather than uniform reprogramming after bifidobacterial priming. Taken together, these findings suggest that bifidobacteria-derived stimuli are associated with selective modulation of inflammatory responsiveness in moDCs through coordinated changes in immune regulation, metabolic/stress-response pathways, and adhesion/structure-associated programs.

These features are compatible with the broader concept of innate immune memory, in which prior stimulation alters subsequent responsiveness through epigenetic and metabolic reprogramming.^49^^,^^50^ Recent studies have suggested that signals derived from commensal bacteria can imprint memory-like programs in innate immune cells, although evidence for specific taxa remains limited.^28^^,^^30^ In our system, the combination of altered secondary responsiveness, preserved induction of selected surface-markers, and H3K4me1-associated remodeling is consistent with a tolerance-leaning interpretation of altered secondary inflammatory responsiveness, rather than with a model of uniform terminal maturation or global immune unresponsiveness. Further support for this interpretation will require clarification of the durability, reversibility, and broader functional scope of this state.

Our study also provides insight into the stage at which bifidobacterial signals may act. We initially performed priming at the monocyte stage because gut-derived microbial components and metabolites may enter the circulation and condition peripheral myeloid cells before or during differentiation.^3^^,^^4^^,^^5^ However, priming of fully differentiated moDCs also attenuated subsequent inflammatory responses, suggesting that the response-modulating effect of bifidobacteria is not restricted to an early differentiation window. These findings raise the possibility that bifidobacterial signals can affect peripheral myeloid cells across multiple differentiation or activation states.

The present study focused on lysate-derived components rather than live bacteria. While this approach supports a role for non-viable bacterial components in modulating moDC responsiveness, it does not exclude additional contributions from metabolites produced by live bifidobacteria, which should be addressed in future studies.^32^

Restrained secondary inflammatory responsiveness may contribute to immune homeostasis during repeated exposure to microbial signals. Excessive innate immune activation or maladaptive training has been implicated in chronic inflammatory disorders,^51^ whereas the mechanisms that support a balanced low-inflammatory state in healthy individuals remain less well understood. Our findings raise the possibility that bifidobacterial exposure may be linked to a lower inflammatory response tone in human monocyte-derived cells.

In conclusion, our findings link greater gut *Bifidobacterium* abundance with attenuated inflammatory responsiveness of human moDCs and show that bifidobacterial priming is accompanied by transcriptomic and H3K4me1-associated changes consistent with a selectively reprogrammed low-inflammatory state *in vitro*. These results provide a basis for further investigation into how commensal bifidobacteria shape inflammatory responsiveness in human monocyte-derived cells through selective reprogramming of their response state.

### Limitations of the study

Nonetheless, several limitations warrant attention. First, our study integrates an observational human cohort analysis with *in vitro* assays and therefore does not establish a direct causal link between gut *Bifidobacterium* abundance and immune responsiveness. While our use of human-derived immune cells enhances translational relevance, it remains unclear whether comparable immune reprogramming occurs under physiological conditions. Thus, future studies using paired samples from the same donors, as well as *in vivo* or intervention-based approaches, will be required to test the causal relationships suggested by these findings. Second, although sex and age, as well as selected dietary variables assessed by a brief self-administered diet history questionnaire, were considered, other background factors potentially relevant to immune responsiveness, including clinical laboratory parameters, were not collected or comprehensively evaluated, and residual confounding cannot be excluded. Third, although moDCs were used as a model, *in vitro* monocyte-to-moDC differentiation does not fully recapitulate dendritic-cell development or dendritic-cell subset heterogeneity *in vivo*. It therefore remains to be determined whether bifidobacteria induce similar effects in primary dendritic-cell subsets or other systemic cell populations. Fourth, while we assessed cytokine production as an outcome, additional functional readouts—such as phagocytosis, antigen presentation, and T cell modulation—could enrich understanding of DC function under bifidobacterial priming. Finally, more precise dissection of bifidobacterial molecular triggers that drive the observed remodeling in moDCs is needed, as are investigations into immediate changes following priming and rechallenge.

## Resource availability

### Lead contact

Further information and requests for reagents can be addressed to and will be fulfilled by the Lead Contact Sayaka Ishihara (sayaka-ishihara125@morinagamilk.co.jp).

### Materials availability

This study did not generate new unique reagents.

### Data and code availability

All data generated or analyzed during this study are included in this published article and its online supplemental information files. The raw 16S rRNA gene sequences have been deposited in the DNA Data Bank of Japan (DDBJ) Sequence Read Archive with the following identifiers: DDBJ Sequence Read Archive: DRR630943-DRR630992, BioProject: PRJDB19950, BioSample: SAMD00872373-SAMD00872422. Shotgun metagenomic sequencing data have been deposited in the DDBJ Sequence Read Archive: DRR1045172-DRR1045221, BioProject: PRJDB42186. RNA-seq and CUT&Tag data have been deposited in the Genomic Expression Archive with the following identifiers: Genomic Expression Archive: E-GEAD-901, E-GEAD-902. This paper does not report original custom code. Any additional information required to reanalyze the data reported in this paper is available from the lead contact upon reasonable request.

## Acknowledgments

We extend our deepest gratitude to all the participants, Dr. Tominaga, and the staff at Tominaga Internal Medicine Clinic for their invaluable cooperation in this study. We also wish to acknowledge the significant contributions of KY, AK, CY and KY for their assistance.

## Author contributions

Conceptualization: S.I., T.N., and T.O.; investigation: S.I., T.N., R.M., E.M., and A.S.; methodology: S.I., T.N., R.M., and T.O.; visualization: S.I., T.N., R.M., and T.O.; formal analysis: S.I., T.N., and T.O.; data curation: H.K. supervision: Y.H., M.T., and T.O.; project administration: M.T. and T.O.; writing – original draft: S.I., T.N., and R.M.; writing – review and editing: S.I., T.N., and T.O.; all authors read and approved the manuscript for publication.

## Declaration of interests

All authors are employees of Morinaga Milk Industry Co., Ltd., which has several probiotic products marketed worldwide. SI and TN are inventors on patents related to the technology discussed in this manuscript, which may earn royalties if the technology is commercialized.

## Declaration of generative AI and AI-assisted technologies in the writing process

During the preparation of this work, the authors used AI-assisted tools to support language editing. After using these tools, the authors reviewed and edited the content as needed and take full responsibility for the content of the publication.

## STAR★Methods

### Key resources table

**Table:** 

REAGENT or RESOURCE	SOURCE	IDENTIFIER
**Antibodies**		

Rabbit polyclonal anti-H3K4me1	Active Motif	Cat#39299; RRID: AB_3216390
Rabbit polyclonal anti-H3K27ac	Active Motif	Cat#39134; RRID:AB_2722569
Rabbit polyclonal anti-H3K27me3	Active Motif	Cat#39156; RRID:AB_2636821
BV480 anti-human CD14	BD	Cat#566141; RRID:AB_2739539
Brilliant Violet 421™ anti-human CD16 Antibody	BioLegend	Cat#302038; RRID:AB_2561578
Brilliant Violet 421™ anti-human CD40 Antibody	BioLegend	Cat#334332; RRID:AB_2564211
BB700 anti-human HLA-DR	BD	Cat#566480; RRID:AB_2744477
PE anti-human CD209 (DC-SIGN) Antibody	BioLegend	Cat#330106; RRID:AB_1134052
APC anti-human CD86 Antibody	BioLegend	Cat#374208; RRID:AB_2721449
PE/Cyanine7 anti-human CD83 Antibody	BioLegend	Cat#305325; RRID:AB_2561774
PE/Cyanine7 anti-human CD197 (CCR7) Antibody	BioLegend	Cat#353226; RRID:AB_11126145
BD Pharmingen™ Human BD Fc Block	BD	Cat#564220; RRID:AB_2869554

**Bacterial and virus strains**		

*Bifidobacterium longum* subsp. *longum* BB536	Morinaga Milk Industry	N/A
*Bifidobacterium breve* MCC1274	Morinaga Milk Industry	N/A
*Lacticaseibacillus paracasei* MCC1849	Morinaga Milk Industry	N/A
*Bifidobacterium adolescentis* JCM1275	JCM	JCM1275
*Bifidobacterium breve* JCM1192	JCM	JCM1192
*Bifidobacterium longum* subsp. *longum* JCM1217	JCM	JCM1217
*Bifidobacterium pseudocatenulatum* JCM1200	JCM	JCM1200
*Faecalibacillus faecis* JCM32257	JCM	JCM32257
*Faecalibacillus intestinalis* JCM34082	JCM	JCM34082
*Bacteroides uniformis* JCM5828	JCM	JCM5828
*Phocaeicola vulgatus* JCM5826	JCM	JCM5826

**Biological samples**		

Blood samples	This paper	N/A
Fecal samples	This paper	N/A
PBMCs	STEMCELL Technologies	Cat#ST-70025

**Chemicals, peptides, and recombinant proteins**		

human recombinant GM-CSF	R&D Systems	Cat#215-GM-050
human recombinant IL-4	R&D Systems	Cat#204-IL-050
eBioscience™ Fixable Viability Dye eFluor™ 780	Invitrogen	Cat#65086514
LPS	Sigma-Aldrich	Cat#L2630

**Critical commercial assays**		

EasySep^TM^ human monocyte enrichment kit	STEMCELL Technologies	Cat#19059
Luminex® multiplex assays	R&D Systems	Cat#LXSAHM
CBA human IL-10 flex kit	BD	Cat#558274
CBA human MCP-1 flex kit	BD	Cat#558287
CBA human MIP-1α flex kit	BD	Cat#558325
CBA human IL-1β flex kit	BD	Cat#558279
CBA human TNF flex kit	BD	Cat#558273
CBA human IL-6 flex kit	BD	Cat#558276
CBA human IL-8 flex kit	BD	Cat#558277
CBA human IL-12p70 flex kit	BD	Cat#558283
NucleoSpin RNA XS	Macherey-Nagel	Cat#740902.50
Wash Buffer RAW2	Macherey-Nagel	Cat#740359.80
SMART-Seq mRNA LP	Takara Bio	Cat#634768
Nextera XT DNA Library Prep Kit	Illumina	Cat#FC-131-1024
IDT for Illumina - DNA/RNA UD Indexes, Tagmentation	Illumina	Cat#20091654
NovaSeq X Series 25B Reagent Kit	Illumina	Cat#20104706
CUT&Tag-IT® Assay Kit	Active Motif	Cat#53160
NextSeq 1000/2000 P1 reagent kit	Illumina	Cat#20100981
ThruPLEX DNA-Seq Kit	Takara Bio	Cat#R400674
Unique Dual Index Kit	Takara Bio	Cat#634756
D1000 ScreenTape	Agilent Technologies	Cat#5067-5582
D1000 Reagents	Agilent Technologies	Cat#5067-5583
iSeq 100 i1 Reagent v2	Illumina	Cat#20031371
NovaSeq X Series 10B Reagent Kit (300-cycle)	Illumina	Cat#20085594
NovaSeq X Series 25B Reagent Kit (300-cycle)	Illumina	Cat#20104706

**Deposited data**		

16S rRNA gene sequence data	This paper	DDBJ Sequence Read Archive: DRR630943-DRR630992; Bioproject: PRJDB19950; BioSample: SAMD00872373–SAMD00872422
RNA-seq data	This paper	Genomic Expression Archive: E-GEAD-901
CUT&Tag data	This paper	Genomic Expression Archive: E-GEAD-902
Shotgun metagenome sequencing	This paper	DDBJ Sequence Read Archive: DRR1045172-DRR1045221; BioProject: PRJDB42186

**Software and algorithms**		

R (v.4.3.1)	R Project	https://www.r-project.org
QIIME2 (v.2022.8)	QIIME2	https://qiime2.org
DADA2	Bioconductor	https://bioconductor.org/packages/release/bioc/html/dada2.html
NovaSeq X Plus Control Software (v.1.2.0)	Illumina	N/A
DRAGEN Bio-IT Platform (v.4.3.6)	Illumina	N/A
RSeQC (v.2.6.4)	RSeQC	https://rseqc.sourceforge.net
DEseq2 (v.1.28.0)	Bioconductor	https://bioconductor.org/packages/release/bioc/html/DESeq2.html
bcl2fastq2 (v2.20.0)	Illumina	https://jp.support.illumina.com/downloads/bcl2fastq-conversion-software-v2-20.html
BWA (v0.7.12)	BWA	https://github.com/lh3/bwa
picard (v.3.1.0)	Broad Institute	https://broadinstitute.github.io/picard/
bedtools (v2.31.1)	bedtools	https://github.com/arq5x/bedtools2
wigToBigWig (v4)	UCSC Genome Browser	https://hgdownload.soe.ucsc.edu/admin/exe/
MACS (v2.1.0)	MACS2	https://github.com/macs3-project/MACS
Subread (v1.5.2)	Subread	https://subread.sourceforge.net
Metascape	Metascape	https://metascape.org
BD® CBA Analysis Software (v1.0.2)	BD Biosciences	https://www.bdbiosciences.com/ja-jp/products/reagents/immunoassays/cba
Hostile (v2.0.2)	Hostile	https://github.com/bede/hostile
fastp (v1.1.0)	fastp	https://github.com/opengene/fastp
MetaPhlAn (v4.2.4)	MetaPhlAn	https://github.com/biobakery/metaphlan
CHOCOPhlAnSGB database	MetaPhlAn	https://github.com/biobakery/MetaPhlAn
FlowJo (v10.10.1)	BD biosciences	https://www.flowjo.com

### Experimental model and study participant details

#### Ethics statement

This study was conducted in compliance with the Declaration of Helsinki (Fortaleza, revised in 2013) and the Ethical Guidelines for Life Sciences and Medical Research Involving Human Subjects (Ministry of Education, Culture, Sports, Science and Technology; Ministry of Health, Labor and Welfare; and Ministry of Economy, Trade and Industry Notification No. 1, 2021). Approval was obtained from the Research Ethics Committee of the Japan Conference of Clinical Research (JCCR), reference number 504 (date 18 August 2023). This trial was registered at the University Hospital Medical Research Network under UMIN000052004. Before the start of the study, all participants were thoroughly informed of its purpose and procedures. Written informed consent was obtained from all the participants.

#### Participants

Japanese adults were recruited and screened for eligibility. Inclusion required that participants be healthy and at least 18 years old. The exclusion criteria were as follows.

1. Current treatment for malignant tumors or respiratory, liver, kidney, heart, lung, digestive, blood, endocrine, or metabolic diseases or a serious medical history of these conditions.
2. Known drug or severe food allergies, or a history of such allergies.
3. Pregnancy or breastfeeding.
4. Autoimmune disorders.
5. Planned use of medications potentially affecting immune function or gut microbiota (e.g., antiallergic agents, anti-inflammatory agents, antibiotics, gastrointestinal medications, diabetes medications) from the time of consent until the end of the study.
6. Planned vaccinations (e.g., influenza, COVID-19) from the time of consent until the end of the study.
7. Any condition deemed unsuitable by the principal investigator or study physician on background, physical or infection test results.

#### Human samples

Fecal and peripheral blood samples were collected within one day of each other to facilitate simultaneous assessment of immune parameters and gut microbiota composition. Immediately after collection, fecal samples were frozen at −80°C. Peripheral blood samples were used to isolate monocytes for downstream differentiation into moDCs. For the *in vitro* priming–differentiation–rechallenge experiments assessing altered secondary responsiveness, cryopreserved PBMCs from de-identified healthy adult donors were purchased from STEMCELL Technologies. Both male and female donors were included.

#### Bacterial strains and culture

*Bifidobacterium longum* subsp*. longum* BB536 (*B. longum*), *Bifidobacterium breve* MCC1274 (*B. breve*), and *Lacticaseibacillus paracasei* MCC1849 (*L. paracasei*) were obtained from the Morinaga Culture Collection (Morinaga Milk Industry). *Bifidobacterium longum* subsp. *longum* JCM1217 (BL or *B. longum*), *Bifidobacterium pseudocatenulatum* JCM1200 (BP or *B. pseudocatenulatum*), *Bifidobacterium adolescentis* JCM1275 (BA or *B. adolescentis*), *Bifidobacterium breve* JCM1192 (BB or *B. breve*), *Faecalibacillus faecis* JCM32257 (FF or *F. faecis*), *Faecalibacillus intestinalis* JCM34082 (FI or *F. intestinalis*), *Bacteroides uniformis* JCM5828 (*B. uniformis*) and *Phocaeicola vulgatus* JCM5826 (*P. vulgatus*) were obtained from the Japan Collection of Microorganisms (RIKEN BRC). *Bifidobacterium* strains and *Lacticaseibacillus* strain were cultured in MRS broth (Difco Laboratories), and *Faecalibacillus*, *Bacteroides* and *Phocaeicola* strains were cultured in GAM broth (Nissui), both supplemented with 0.05% (w/v) L-cysteine hydrochloride (Kanto Chemical), for 16 h at 37°C under anaerobic conditions using an AnaeroPack (Mitsubishi Gas Chemical).

### Method details

#### Preparing heat-killed bacteria

Bacterial cells were harvested by centrifugation at 8,000 × g for 5 min, washed twice in distilled water (Wako), and resuspended in 0.22 μm-filtered distilled water. To inactivate the cells, the suspensions were heated at 90°C for 15 min. The final concentration was adjusted to 10^9^ cells/mL, with cell counts determined by hemocytometer.

#### Generation of bacterial lysate supernatants from *Bifidobacterium* and *Faecalibacillus*

Bacterial cells were harvested as above and resuspended at 1× 10^9^ cells/mL. For lysis, 0.3 g of 0.1 mm glass beads was added to 0.5 mL aliquots of each bacterial suspension. The mixture was mechanically disrupted by bead beating using a FastPrep-24 5G cell disruptor (Funakoshi) at a speed of 5.0 for 45 s at room temperature, followed by 5 min on ice. This process was repeated three times. The lysates were then centrifuged at 13,000 × g for 5 min at 4°C, and the supernatants were filtered through a 0.22 μm filter and stored at −80 °C until use. The protein concentration was determined using the Pierce BCA protein Assay kit (ThermoFisher Scientific).

#### Participant-derived PBMC and monocyte isolation, moDC generation, and stimulation

PBMCs were isolated by using Vacutainer CPT tubes (BD) according to the manufacturer’s instructions. Monocytes were subsequently enriched from PBMCs by negative selection with an EasySep human monocyte enrichment kit (STEMCELL Technologies) according to the manufacturer’s instructions. Flow cytometry confirmed that CD14^+^ cells represented >90% of the enriched monocytes.

Isolated monocytes were seeded at 5× 10^5^ cells/ml in 24-well plates (500 μL/well) in complete RPMI-1640 medium supplemented with 100 U/ml penicillin (Sigma‒Aldrich), 10% fetal bovine serum (FBS, Cytiva), 10 ng/mL human recombinant GM-CSF (hrGM-CSF), and 10 ng/mL human recombinant IL-4 (hrIL-4) (both from R&D Systems). Cells were incubated at 37 °C under 5% CO2 for 6 days to generate moDCs. On day 3 or 4, half of the medium was replaced with fresh medium containing hrIL-4 and hrGM-CSF.

Differentiated moDCs were seeded at 1 × 10^5^ cells in 100 μL of RPMI 1640 medium supplemented with 100 U/ml penicillin and 10% FBS in 384-well tissue culture plates (Corning). The cells were stimulated with 1 × 10^6^ particles of heat-killed *B. longum*, *B. breve*, *L. paracasei* or 10 ng/mL LPS from *Escherichia coli* O111:B4 (Sigma‒Aldrich). Unstimulated control cells received medium only. After 24 h at 37°C under 5% CO_2_, supernatants were collected and stored at −80°C.

#### DNA extraction and 16S sequencing

Bacterial DNA was extracted as previously described,^52^ and the V3-V4 region of the 16S rRNA gene was amplified. Paired-end sequencing was performed on the Illumina NextSeq 1000 platform with a NextSeq 1000/2000 P1 reagent kit (600 cycles) (Illumina). Sequences were processed in QIIME2 software (version 2022.8),^53^ including demultiplexing, primer trimming, quality filtering, denoising, and chimera removal via DADA2.^54^ Taxonomy was assigned using the Greengenes2 database (version 2022.10). An average of 26,892 ± 15,774 reads per sample was obtained. For alpha diversity indices (observed features, Shannon entropy, and Faith’s phylogenetic diversity), samples were rarefied to 9,292 sequences per sample, the lowest sequence count among the samples. Beta diversity (weighted UniFrac distances) was assessed using the full dataset.

#### Shotgun metagenomic sequencing

Shotgun metagenomic sequencing was performed using the same fecal DNA samples as those used for 16S rRNA gene profiling. After quality assessment, sequencing libraries were prepared from 100 ng of input DNA using the ThruPLEX DNA-Seq Kit and Unique Dual Index Kit (Takara Bio). Libraries were sequenced on an Illumina NovaSeq X Plus platform to generate 150-bp paired-end reads. Library preparation, shotgun metagenomic sequencing, and primary data processing were performed by Takara Bio Inc.

#### Shotgun metagenomic data analysis

Human host reads were removed from paired-end shotgun metagenomic sequencing data using Hostile v2.0.2, followed by adapter trimming and quality filtering with fastp v1.1.0. Microbial community composition was then profiled with MetaPhlAn 4 (v4.2.4) against the CHOCOPhlAnSGB marker-gene database (mpa_vJan25_CHOCOPhlAnSGB_202503). Species-level relative abundances were extracted from the MetaPhlAn 4 output, and species with a mean relative abundance of ≥1.0% and a prevalence of ≥10% were retained for correlation analysis. For each stimulation condition, Spearman’s rank correlations between species abundance and each moDC cytokine were calculated using all samples with available paired data. *p* values were adjusted within each condition using the Benjamini–Hochberg procedure, and an FDR of <0.05 was considered statistically significant.

#### *In vitro* priming, differentiation, and rechallenge of moDCs

Monocytes isolated from PBMCs purchased from STEMCELL Technologies were seeded at 5× 10^5^ cells/ml in 24-well plates (with 500 μL/well) or 6-well plates (with 2 mL/well) in complete RPMI-1640 medium containing 100 U/ml penicillin and 10% FBS. They were primed for 24 h with bacterial lysate supernatants of BL, BP, FF, or FI, standardized to a protein concentration of 2 μg/mL, 10 ng/mL LPS as a reference priming condition, or maintained in RPMI as the unprimed control (UC). After priming, cells were washed twice with warm RPMI-1640, and fresh complete medium containing 10 ng/mL hrIL-4 and 10 ng/mL hrGM-CSF was added. On day 4 or 5, half of the medium was replaced with fresh medium containing hrIL-4 and hrGM-CSF. On day 7, the differentiated moDCs were either stimulated again with heat-killed *B. longum*, *B. breve*, *L. paracasei*, *F. intestinalis*, *F. faecis*, *B. uniformis* or *P. vulgatus* or 10 ng/mL LPS. After 24 h, supernatants were collected and stored at −80°C. For downstream CUT&Tag or RNA-seq assays, moDCs on day 7 were harvested as follows: (i) for CUT&Tag, cells were resuspended in CELLBANKER 1plus (Takara) and stored at −80°C; (ii) for RNA-seq, cells were washed with RPMI-1640, culture medium was removed, and cells were stored at −80°C until use.

#### Flow cytometric analysis

Flow-cytometric analysis was performed to assess moDC differentiation, and surface marker expression before and after LPS rechallenge. Monocytes left unprimed or primed with bacterial lysate supernatants for 24 h were then cultured for 6 additional days in the presence of GM-CSF and IL-4. Cells collected on day 7 were analyzed to evaluate differentiation into moDCs and their baseline phenotypic status before rechallenge. For post-rechallenge analysis, day-7 moDCs were stimulated with LPS for 24 h and analyzed on day 8. Day-7 UC-, BL-, BP-, FI-, and FF-moDCs were analyzed for CD14, CD209, CD83, CD86, CD40, HLA-DR, CCR7, and viability, whereas day-8 cells after LPS rechallenge were analyzed for HLA-DR, CD86, CD40, and viability. Cells were washed with PBS and stained with Fixable Viability Dye eFluor 780 (Invitrogen) for 15 min to exclude dead cells. After washing, cells were incubated with Fc receptor blocking reagent (BD Biosciences) for 15 min, followed by surface staining for 30 min with fluorochrome-conjugated antibodies against human CD14, CD209, CD83, CD86, CD40, HLA-DR, and CCR7 (BD Biosciences or BioLegend). After staining, cells were washed, fixed, and acquired on a BD FACSLyric flow cytometer. Compensation was performed using single-stained controls. Data were analyzed using FlowJo software (v10.10.1). Cells were gated sequentially on FSC-A/SSC-A, singlets (FSC-A versus FSC-H), and live cells. Surface-marker expression was evaluated within the gated population, with positivity thresholds defined based on unstained controls. Results are presented as the percentage of positive cells and/or median fluorescence intensity (MFI). For post-rechallenge analyses, changes in HLA-DR, CD86, and CD40 expression were additionally calculated as ΔMFI (day 8 minus day 7).

#### Priming and rechallenge of fully differentiated moDCs

Isolated monocytes were cultured in the presence of 10 ng/mL hrGM-CSF and 10 ng/mL hrIL-4 for 6 days to differentiate into moDCs. On day 6, the moDCs were harvested and primed for 24 h with 2 μg/mL protein-standardized lysate supernatants of BL or BP or maintained in RPMI as unprimed control. After priming, cells were washed twice with warm RPMI-1640, and fresh complete medium containing 10 ng/mL hrIL-4 and 10 ng/mL hrGM-CSF was added. On day 10 or 11, half of the medium was replaced with fresh medium containing hrIL-4 and hrGM-CSF. On day 13, the moDCs were either stimulated again with heat-killed *B. longum* or 10 ng/mL LPS. After 24 h, supernatants were collected and stored at −80°C.

#### Cytokine assays

Cytokine concentrations (IL-1Ra, IL-10, CCL2, CCL3, IL-1β, TNFα, IL-6, IL-8, and IL-12) were measured via Luminex multiplex assays (R&D Systems) or cytometric bead arrays (BD), following the manufacturers’ instructions. For Luminex multiplex assays, data analysis was performed using the Bio-Plex 200 system. For cytometric bead arrays, data analysis was performed using BD FACSLyric and BD CBA Analysis Software (v.1.0.2).

The cytokine panel was defined *a priori* as a focused readout to capture key functional features of human moDC responses to microbial stimulation. Specifically, it included pro-inflammatory cytokines (IL-1β, TNFα, IL-6, and IL-12), regulatory mediators (IL-10 and IL-1Ra), and chemokines involved in immune cell recruitment and activation (CCL2, CCL3, and IL-8). In the cohort-based moDC stimulation assays, a broader panel was used to capture inter-individual variability across multiple response dimensions. In contrast, the priming–rechallenge experiments employed a reduced panel of representative cytokines to assess reproducible changes in secondary inflammatory responsiveness.

#### RNA extraction and sequencing

Total RNA was extracted using NucleoSpin RNA XS (Macherey-Nagel) according to the manufacturer’s instructions. RNA purity, concentration, and integrity were evaluated with an Agilent TapeStation or a BioAnalyzer (Agilent Technologies). RNA libraries were prepared using an SMART-Seq mRNA LP Kit (Takara Bio) and a Nextera XT DNA Library Prep Kit (Illumina), then sequenced on an Illumina NovaSeq X Plus (2 × 150 bp). For the *B. longum*-primed group, only two biological replicates were analyzed due to financial constraints associated with outsourced RNA sequencing. No samples were excluded as outliers.

#### RNA-seq data analysis

Raw data were base-called using NovaSeq X Plus Control Software (v.1.2.0) and processed with the DRAGEN Bio-IT Platform (v.4.3.6) at default settings. Reads were aligned to the human reference genome GRCh38. RNA-seq quality was assessed using RSeQC (v.2.6.4). DEGs were identified via Deseq2 (v.1.28.0), applying an adjusted *p*-value cutoff of 0.05 and log2 fold change threshold of ±1.

#### CUT&Tag chromatin profiling

CUT&Tag was performed by Active Motif. First, cells were incubated overnight with concanavalin A beads and 1 μL of the primary antibody (anti-H3K4me1, anti-H3K27ac, or anti-H3K27me3; Active Motif). After washing and secondary antibody incubation (anti-rabbit 1:100), cells underwent tagmentation at 37°C with protein-A-Tn5. Reactions were stopped by adding EDTA, SDS and proteinase K at 55°C. DNA was extracted, purified, PCR-amplified, barcoded, and cleaned with SPRI beads (Beckman Coulter). Libraries were quantified and sequenced on an Illumina NextSeq 550 (PE38). For the *B. longum*-primed group, only two biological replicates were analyzed due to financial constraints associated with outsourced CUT&Tag assay. No samples were excluded as outliers.

#### CUT&Tag data analysis

Data were processed using the nf-core/cutandrun pipeline. The bcl2fastq2 (v2.20.0) handled base-call data and demultiplexing. Paired-ended reads were aligned to hg38 using BWA (v0.7.12) with default settings. Duplicates were removed via picard (v.3.1.0). Bed and bigWig files were generated using bedtools (v2.31.1) and wigToBigWig (v4). Peaks were called with MACS (v2.1.0) using a threshold of p < 1e−7 and the –nomodel option. Blacklisted regions (ENCODE) were removed. Signal maps and peak locations were input into Active Motif’s proprietary software to generate Excel files containing sample comparisons, peak metrics, locations and gene annotations. For differential analysis, reads in merged peak regions were quantified using Subread (v1.5.2), and replicates comparisons were conducted via DESeq2 (v1.28.0) with an adjusted *p*-value cutoff of 0.05 and log2 fold change threshold of ±0.5.

#### Integrated analysis of RNA-seq and CUT&Tag data

A gene-centric approach was used to merge DEGs with gene-annotated CUT&Tag peaks. Matching was performed by gene name, using the R merge function to identify overlapping events.

#### GO and KEGG pathway analysis

GO terms and KEGG pathways enrichment for DEGs and dynamic H3K4me1 peaks were performed using Metascape.^55^ The analyses focused on genes or peaks commonly up- or downregulated in BL- and BP-moDCs. Biological processes and KEGG pathways were ranked by adjusted *p*-value (top 20 displayed).

### Quantification and statistical analysis

Dunnett’s test or Tukey’s test were used to compare multiple groups while paired comparisons were analyzed using a paired *t* test. Spearman’s rank correlation analysis was conducted to assess the relationships between the variables. *p*-values were corrected for multiple comparisons using the Benjamini–Hochberg (BH) method. For beta diversity analyses, nested PERMANOVA with 999 permutations was conducted on Bray‒Curtis dissimilarities using the Adonis test in the vegan R package (v2.6.2). Error bars in figures indicate standard deviation. *p* values of less than 0.05 were considered significant. Analyses were carried out in R (ver. 4.3.1). The number of replicates and the statistical tests used are indicated in each figure legend.

### Additional resources

This study was registered with the University Hospital Medical Information Network Clinical Trials Registry under UMIN000052004.
