# Supplementary material for: Gut commensal bifidobacteria are associated with restrained inflammatory reprogramming in human monocyte-derived dendritic cells
Source: iScience. 2026 Jun 30;29(7):116583. doi: 10.1016/j.isci.2026.116583 (PMC13378373; doi:10.1016/j.isci.2026.116583)
Supplement: Document S1. Figures S1–S5 and Table S1 [file mmc1.pdf]

iScience, Volume *29*

Supplemental information

Gut commensal bifidobacteria are associated with restrained inflammatory reprogramming in human monocyte-derived dendritic cells

Sayaka Ishihara, Tatsuki Nishimura, Riko Mishima, Eri Mitsuyama, Akira Sen, Hiroki Kaneko, Yukihiro Hishida, Miyuki Tanaka, and Toshitaka Odamaki

Supplemental information

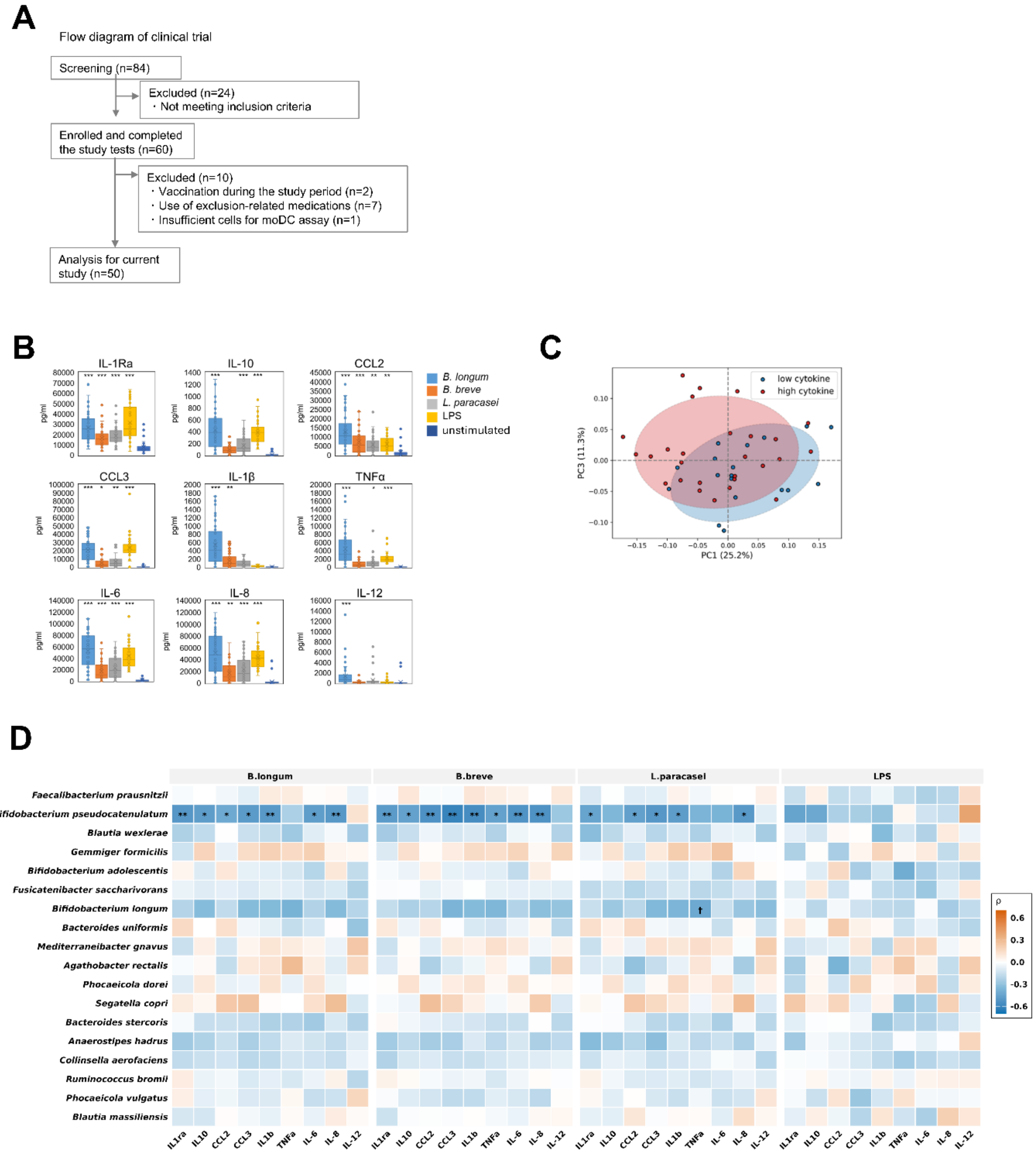

Figure S1. Additional cohort-level analyses of cytokine responsiveness and gut microbiota composition

(A) Flow diagram showing participant inclusion and exclusion and the number of subjects analysed. Ten participants were excluded from the final analysis after post-study quality

review, due to protocol deviations (e.g., vaccination during the study period) or technical limitations (e.g., insufficient cell yield for moDC assays). (B) Each box spans the interquartile range (IQR), the lines within the boxes represent the median and whiskers extend to the lowest and highest values within 1.5 times IQR. Points beyond the whiskers are outliers. Data are shown for *B. longum* (n = 50), *B. breve* (n = 49), *L. paracasei* (n = 48), LPS (n = 45), and unstimulated control (n = 50). One-way ANOVA followed by Dunnett's multiple comparisons test was used to compare each mean to the unstimulated control (*p < 0.05, **p < 0.01, ***p < 0.001). (C) A principal coordinate analysis (PCoA) plot based on weighted UniFrac distances, comparing the gut microbiota of Group 1 (red) and Group 2 (blue) (n=45). Ellipses represent the 90% confidence intervals; the percentages in parentheses represent the proportion of variation explained by each PCoA axis. Statistical significance between the two groups was assessed using permutational multivariate analysis of variance (PERMANOVA) ($p = 0.025$). (D) Spearman's rank correlation between moDC cytokine and chemokine responses to the indicated stimuli and the relative abundance of gut bacterial species identified by shotgun metagenomic sequencing (restricted to species with mean relative abundance >1% and prevalence ≥10%). Colors represent correlation coefficients (red, positive; blue, negative). Data are shown for *B. longum* (n = 50), *B. breve* (n = 49), *L. paracasei* (n = 48), and LPS (n = 45). Statistical significance was assessed using two-sided Spearman's rank correlation with Benjamini–Hochberg correction for multiple testing and is indicated in the heatmap (†p < 0.1, *p < 0.05). Correlation coefficients and corresponding raw and adjusted p values are provided in Table S5.

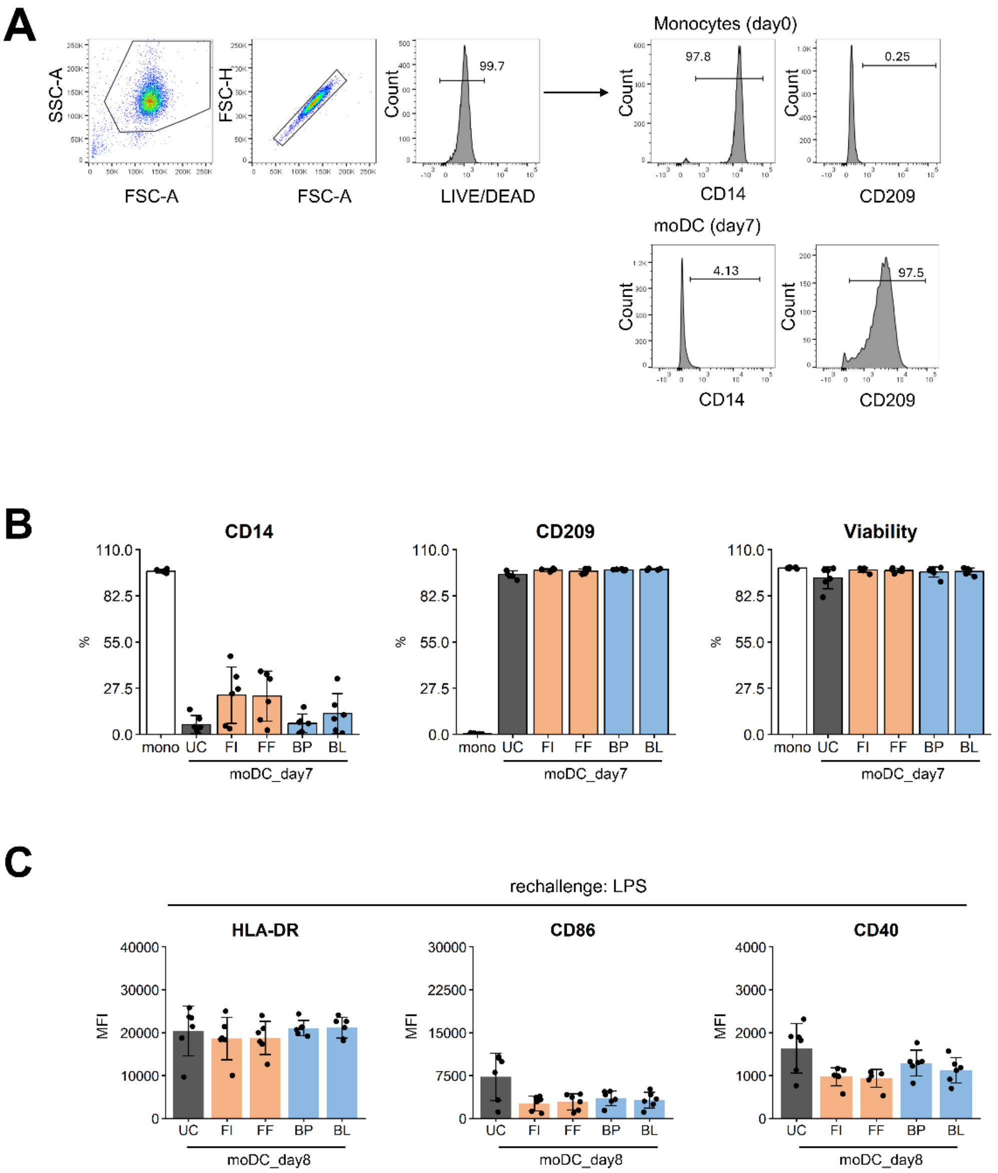

Figure S2. Flow-cytometric validation of moDC differentiation and phenotypic analyses. (A) Representative gating strategy for flow-cytometric analysis. Cells were gated sequentially on FSC-A/SSC-A, singlets, and live cells, followed by assessment of CD14 and CD209 expression. Representative histograms for monocytes at day 0 and moDCs at day 7 are shown. (B) Validation of monocyte-to-moDC differentiation at day 7. CD14 and CD209 expression were assessed in day-0 monocytes and in day-7 moDCs generated under unprimed control (UC), *F. intestinalis* (FI), *F. faecis* (FF), *B. pseudocatenulatum* (BP), and *B. longum* (BL) priming conditions. Viability is also shown. Data are presented as mean ± SD with individual data points. (C) Surface-marker expression on day 8 after LPS rechallenge.

Median fluorescence intensity of HLA-DR, CD86, and CD40 is shown for UC-, FI-, FF-, BP-, and BL-moDCs. Data are presented as mean ± SD with individual data points. Statistical analysis was performed using Dunnett's test with Benjamini–Hochberg correction for multiple testing; no statistically significant differences were observed among conditions.

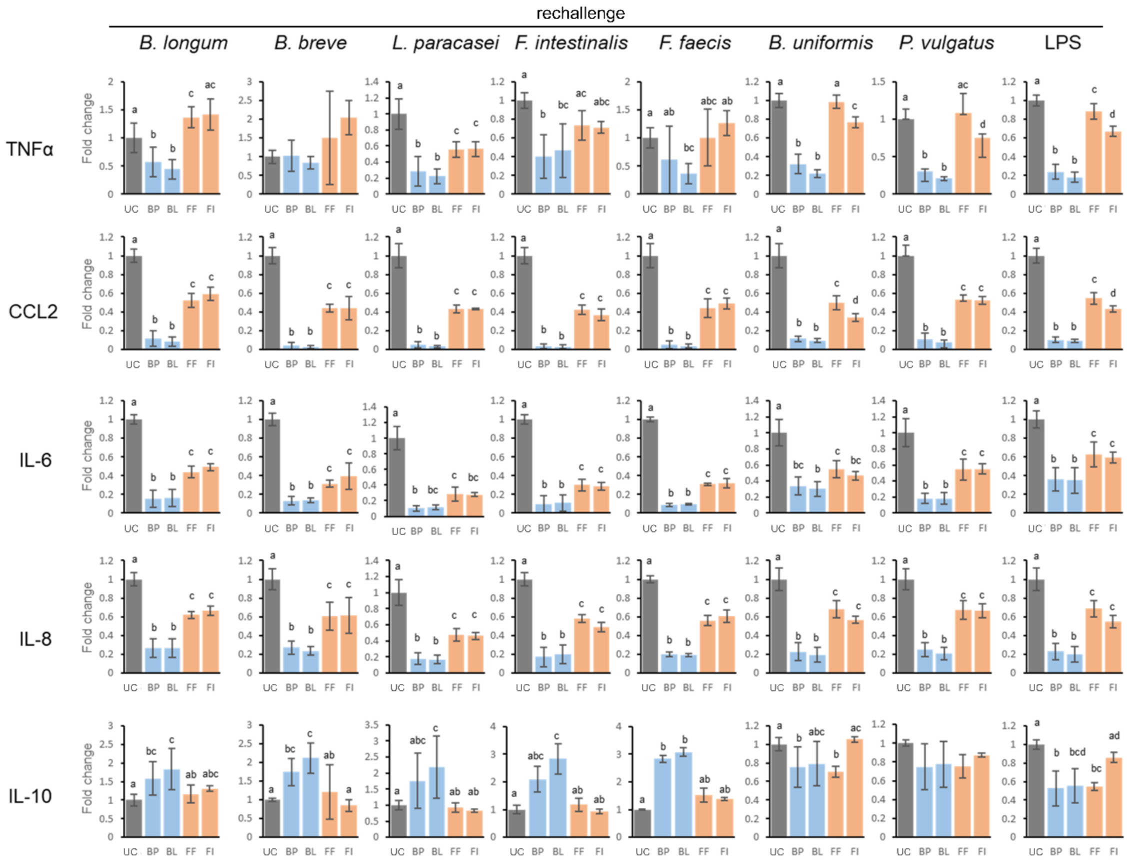

Figure S3. Cytokine and chemokine responses of primed moDCs upon rechallenge. Cytokine and chemokine production by moDCs primed with bacterial lysates from *Bifidobacterium* or *Faecalibacillus* species and subsequently rechallenged, shown as fold change relative to control (n = 3-8). Data are presented as mean ± SD. Statistical analysis was performed using Tukey's test (for pairwise comparisons among all treatment groups). Different lowercase letters indicate significant differences at adj. $p < 0.05$. Each abbreviation indicates the bacteria used for priming: FI for *F. intestinalis*, FF for *F. faecis*, BL for *B. longum*, BP for *B. pseudocatenulatum* and UC for unprimed control.

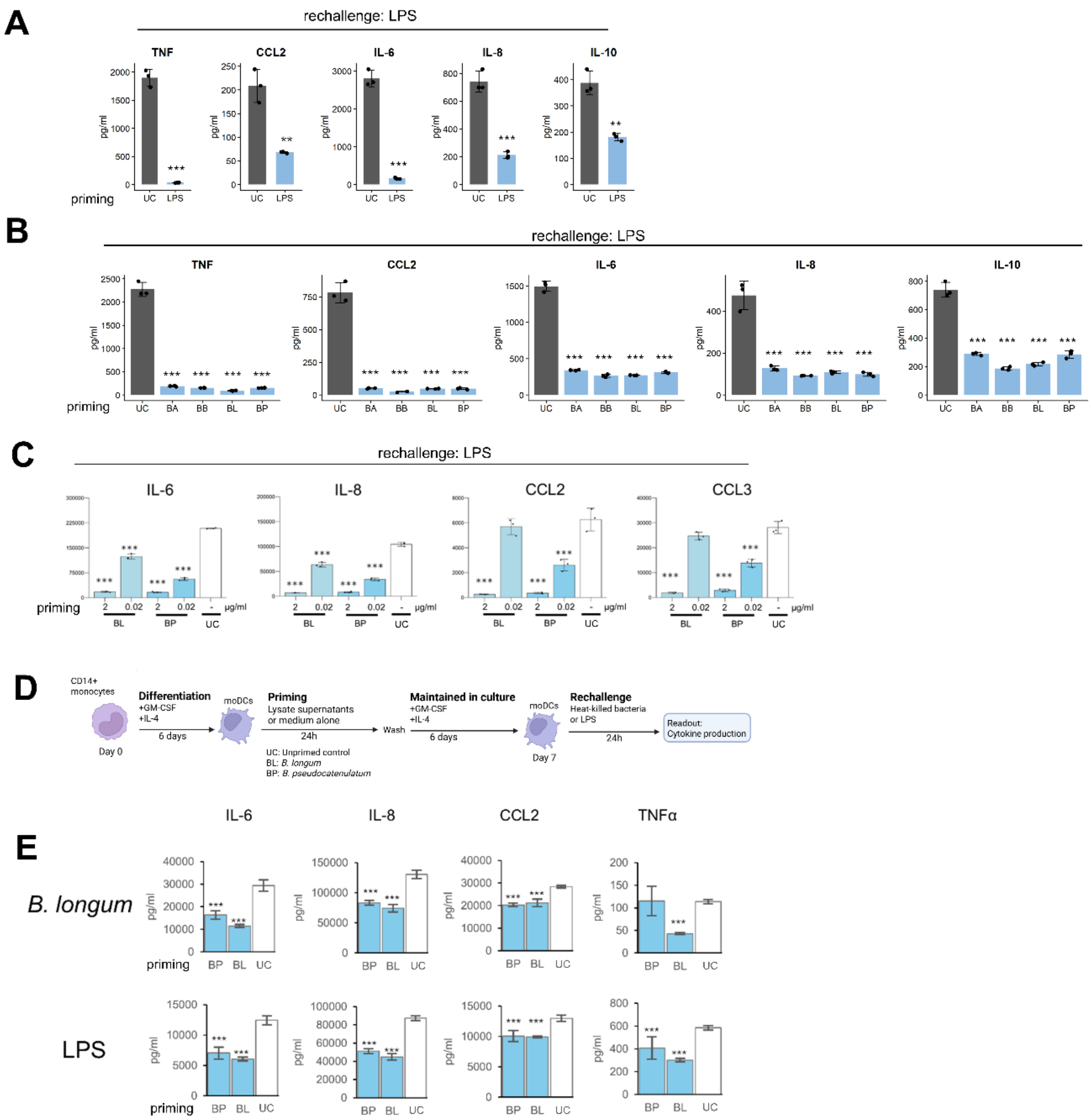

Figure S4. Additional analyses of inflammatory responses upon rechallenge following priming.

(A) Reference LPS-tolerance condition. Cytokine and chemokine responses after LPS rechallenge are shown for unprimed control moDCs (UC) and LPS-primed moDCs. Monocytes were primed with LPS or medium alone, differentiated into moDCs, and rechallenged with LPS. (B) Cytokine and chemokine responses after LPS rechallenge in moDCs primed with additional *Bifidobacterium* species. In addition to *B. longum* (BL) and *B. pseudocatenulatum* (BP), *B. adolescentis* (BA) and *B. breve* (BB) were evaluated. (C) The suppressive effect of *Bifidobacterium* priming on inflammatory cytokine production by moDCs is dose-dependent. Cytokine and chemokine release decreased with increasing

priming protein concentration. After priming, the cells were stimulated with LPS. (D) Schematic of the experimental design used to assess priming of fully differentiated moDCs. Monocytes were first differentiated into moDCs with GM-CSF and IL-4 for 6 days, then primed for 24 h with bacterial lysate supernatants or medium alone, washed, maintained in culture, and rechallenged with heat-killed bacteria or LPS. The schematic was created with BioRender.com. (E) Cytokine responses of fully differentiated moDCs after priming with BL or BP lysate supernatants and subsequent rechallenge with heat-killed *B. longum* or LPS. All data are presented as mean ± SD with individual data points (n=3 or 4). Statistical analysis was performed using Dunnett's test, comparing each mean to the unprimed control (*$p < 0.05$, **$p < 0.01$, ***$p < 0.001$).

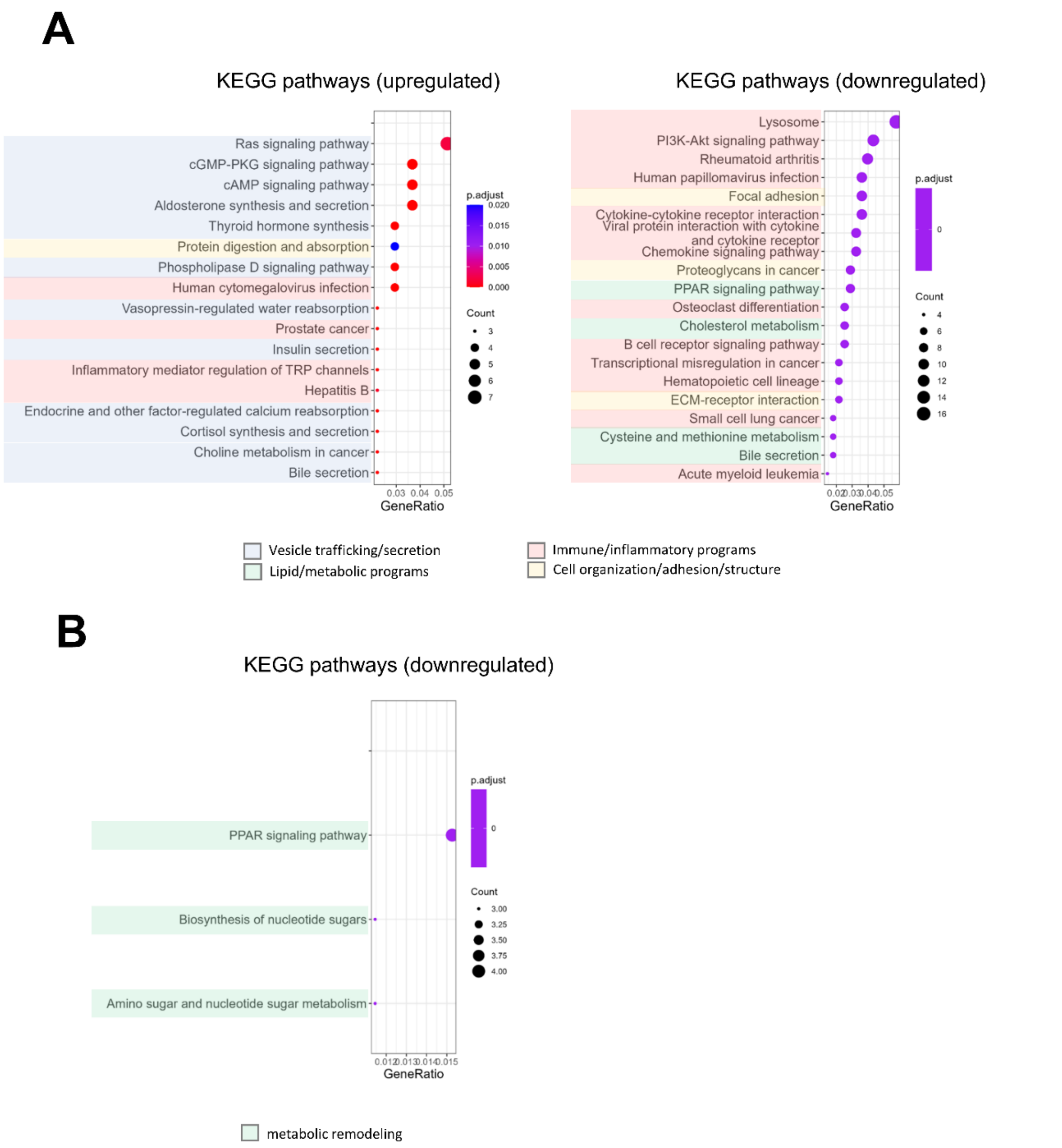

Figure S5. KEGG analysis of DEGs and dynamic H3K4me1 peaks in *Bifidobacterium* primed-moDCs

(A) Dot plots showing the top 20 enriched KEGG pathways altered by *Bifidobacterium* priming in RNA-seq. The analysis considered differentially expressed genes (DEGs) whose expression was consistently upregulated or downregulated in both BL-moDCs and BP-moDCs. Pathways are grouped into broader functional categories. The gene ratio indicates

the proportion of candidate genes within each pathway. Node color reflects the adjusted p-value (adj. $p < 0.05$), and node size corresponds to the number of genes mapped to that pathway. (B) Dot plots showing the top 20 enriched KEGG pathways identified by analyzing significantly up- or downregulated H3K4me1 peaks shared between BP-moDCs and BL-moDCs. Pathways are grouped into broader functional categories. Gene ratio, p-value scale, and node sizes are as in Figure S5A.

Table S1. Baseline characteristics of the subjects.

Characteristics	n=50
Age (years)	39.94 ± 11.72
Sex (male / female)	32/18
BMI	21.46 ± 2.13
